# Dynamic NVH Numerical Analysis of Power Steering in the Presence of Lubricant in the System

**DOI:** 10.3390/ma15072406

**Published:** 2022-03-24

**Authors:** Damian Pietrusiak, Jakub Wróbel, Mateusz Czechowski, Wiesław Fiebig

**Affiliations:** 1 Faculty of Mechanical Engineering, Wroclaw University of Science and Technology, Łukasiewicza 7/9, 50-371 Wroclaw, Poland; jakub.wrobel@pwr.edu.pl (J.W.); wieslaw.fiebig@pwr.edu.pl (W.F.); 2 Nexteer Automotive Poland, NVH Department, Towarowa 6, 43-100 Tychy, Poland; mateusz.czechowski@nexteer.com

**Keywords:** NVH, automotive, finite element analysis (FEA), contact damping, lubricant damping, vibrations, dynamics, Electric Power Steering (EPS), modal analysis, electro-mobility

## Abstract

The ongoing shift towards hybrid and electric vehicles has a strong impact on noise and vibration engineering. New, complex dynamic phenomena are brought to vehicle user attention due to the absence of internal combustion engines and the significant role in vehicle and drive feel perception. This paper presents an FEM (Finite Element Method) dynamic simulation model of an automotive Electric Power Steering assembly. Preliminary modal simulations and experiments as well as field data replication techniques were implemented to identify the phenomena and prepare and validate model components. A full dynamic model of an Electric Power Steering was presented, and fine-tuned including the presence of lubrication at the gear mesh interface. Experimental investigations were conducted alongside FEM simulations for various model setups. Linear and nonlinear contact stiffness models were implemented, as well as contact damping, and simulated at chosen assembly interfaces. The results indicated that in the case of NVH (Noise Vibration and Harshness) analysis of shock/impact originating problems, contact parameters used for static, quasi-static, and low velocity analyses were not applicable. Nonlinear and damped contact stiffness provided better results in such a case.

## 1. Introduction

### 1.1. Evolution of Car Comfort Expectations

**Figure 1:**
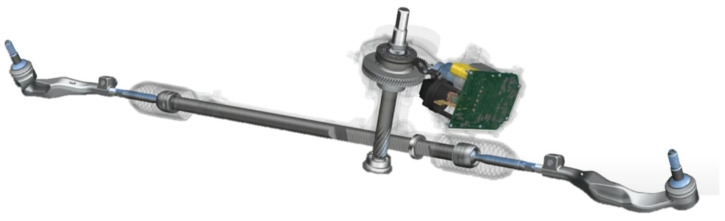
Single Pinion Assist EPS.

Power steering systems used in automotive applications evolved significantly in the last decades. Hydraulic power steering (HPS) was introduced to a production car in the early 1950s and quickly became popular, outperforming geared steering systems. HPS systems utilize hydraulic fluid as a force transmitting medium. Hydraulic pumps provide high pressure fluid supply to the hydraulic assist system, i.e., the rotary valve and hydraulic assist chamber. Hydraulic assist steering systems were fitted to most passenger cars produced in the early 90s until the late 2000s. Heavier vehicles still utilize some form of hydraulic steering assist due to the high power density typical of hydraulic systems. The main disadvantages of HPS are energy loss due to reduced hydraulic system efficiency, leaks, and additional maintenance. The increasing need for low emissions of CO_2_ and overall more efficient vehicles caused a strong shift towards Electric Power Steering (EPS) systems (Figure 1). In the case of EPS systems, the driver input to steering is assisted by an electric motor. The motor input shaft can provide assistance directly at the steering rack or the steering column itself. The main advantages of an EPS over HPS are the reduced fuel consumption [1] and CO_2_ emissions [2], smaller size, and no use of hydraulic fluids. Commonly used types of EPS systems are presented in [3].

Customer expectations regarding vehicles are constantly growing. The importance of vehicle sound quality and the sound development process was emphasized by Schneider et al. [4] in the late 90s. Simultaneously, acoustic improvements of major drive train elements were constantly developed and implemented [5,6,7,8,9,10,11,12]. Otto et al. [13] proposed guidelines for jury evaluation of automotive sounds. Tools for vehicle target sounds and sound quality prediction were investigated by many researchers. Brandl and Biermayer [14] developed software for passenger car interior noise determination based on noise quality maps. Schulte-Fortkamp et al. [15] proposed an Explorative Vehicle Evaluation approach, utilizing a mobile driving simulator for subject-centered target sound determination. Genuit et al. [16] proposed a mobile environment for simulation of the vehicle soundscape. Albert and Schwarz [17] proposed an automated method for automotive NVH phenomena identification. Huang et al. [17] introduced the generalized inverse cascade method designed to solve passenger car NVH problems. 

New noise problems arise as electric and hybrid vehicles generate low background noise in comparison to conventional combustion engine-driven vehicles. Operation at higher frequencies coupled with new lightweight structures of modern vehicles that are easily audible causes new noise and vibration problems previously concealed by the continuous operation internal combustion engine. Chen and Feng [18] presented an overview of NVH problems in hybrid electric vehicles. Putner and Arsic [19] emphasized the differences and new challenges in the experimental investigation of electric vehicle NVH. Lennström et al. [20] studied the perceived annoyance and prominence of tonal components of a purely electric car. Huang et al. [21] proposed the interval analysis method for electric vehicle structure-borne noise problems and presented a case of interior noise reduction by means of EV suspension parameter changes. Qian et al. [22] proposed a pure electric vehicle (PEV) interior sound quality model based on transfer path synthesis (TPS). This research indicated, inter alia, that the PEV interior sound quality is strongly dependent on the road-excited structure-borne noise transmitted by the suspension and the electric drive air-borne noise. Hua et al. [23] summarized recent developments in electric vehicle NVH. 

Improved passenger comfort was the design target in the past and surely will continue to shape the vehicle structure in the future. Lower background pressure levels due the absence of ICE, conventional gearboxes, and improved chassis increase passenger’s perception of other impact-like sounds such as BSR (Buzz, Squeak, and Rattle) [24,25,26], causing annoyance, reduced comfort, and eventually customer complaints. 

### 1.2. Significance of Contact Analysis

Approaching the NVH analyses where the transmissions, gears, etc. are considered, requires inclusion of the dynamics of the system not only in the global scale of the assembly (dynamic characteristics, mode shapes, and frequencies of the components) but also the interactions between the components, which requires a closer look at the contact area and its type because those parameters define the character of local impacts. When analyzing the industrial machinery, where the noise of the machine needs to be limited to the standardized level, additional operators are equipped with hearing protection, and acoustic comfort is not an issue. In that case, the general dynamics approach is in most cases sufficient to protect the machine components against excessive dynamics (i.e., resonance), which can lead to instant damage or progressive wear.

However, in the cases where the acoustic comfort–psychoacoustics is of high significance (NVH automotive), the local impacts, which may not be dangerous to the structural components of the assembly, need to be taken into consideration, while acoustic and vibration emission generated by the local impacts may be the key comfort factor.

The two mentioned mechanisms interact with each other and need to be analyzed simultaneously. The local impact dynamics requires the analysis of several “external” factors, i.e., alternating input forces, clearances between components, and tensioning/mounting/dumping elements, which in the case of global dynamics analysis can be, very often, introduced in a simplified way or simply neglected. However, the most challenging becomes the direct interaction between assembly components, which forces us to analyze the contact type, its stiffness, etc., which is completely negligible when analyzing the global dynamics of the system. Additionally, the factor making the analysis more complex is the presence of lubricants in the contact between components. Their presence have a great influence on the contact behavior, and one can find extreme difficulty in defining the parameters describing them, while there is no direct information about the thickness layer, how it changes under dynamic conditions, and the mechanics of the grease behavior in the specific geometry (local roughness, geometry deviations, etc.), which may vary in tolerance between produced components. The mentioned aspects, which can be specified as tribodynamics [27,28,29], cover the micro-scale tribology and the mixed lubrication where the components can be partially in direct contact and partially lubricated by the oil film. As mentioned, the phenomena taking place at the micro scale and its modelling when performing the analysis in the scale of structural components would excessively complicate the analysis and should be the target of separate investigations. Several aspects of the lubricants and contact stiffness in the automotive industry are presented in the literature [27,28]. 

A preliminary experimental dynamics study of the power steering gear mesh components without lubricant was carried out by the authors of this paper, which revealed the unsteady and non-repetitive results while grease was required for proper smooth and regular operation. A lack of proper lubrication leads to the operation under seizure conditions, and results of the measurements vary at any location of the contact area. Another observation made by the authors, which indicates the lubricant influence on the NVH, was found in rotating machinery where the excessive noise and vibrations were the consequence of the non-uniform application (or aggregation, i.e., due to storage in a horizontal position) of liquid lubricant, which causes the non-uniform friction forces presence or imbalance of the rotating set. The results mentioned above are the qualitative observations of the lubrication influence on vibrations under dynamic conditions. Several aspects of the NVH investigations and the issue of vibration and acoustics in industry are presented in the literature [27,28,29,30]. 

The contact modelling is not only an NVH issue. Billenstein et. al. describes its influence on the topology optimization [31]. Xianwu [32] patented his method for contact stiffness determination based on structural response analysis, and the computational contact mechanics is also an area of development [33,34].

**Figure 2:**
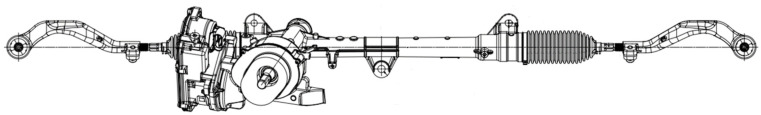
Investigated Electric Power Steering.

Investigations presented in this paper deal with source of the rattle noise in the EPS (Figure 2). The general rattle types in powertrains are idle, drive, and coast, where the drive covers the majority time and due to the load present in the gears has a rather low occurrence [27]. In the case of EPS, the division can be done similarly; however, the idle state is dominant unless driving on curved or uneven roads.

According the reference [35], if analyses concern the relatively slow speed collision between components, which is the consequence of the smooth steering wheel movement, the local contact characteristics are not an issue in modelling. A different situation is when the load is the consequence of an uneven road load while a steering column is fixed. It is a great challenge to develop a structural model whose behavior reflects the vibration phenomena scoping harshness, sound quality, and psychoacoustics. From the point of view of structural engineering only, the influence of a lubricating medium might have been treated as an important but not the leading factor. However, sound quality associated with the lubricating material is the most important factor.

The aim of this paper is to present research targeting the NVH simulations of the components operating in a greased environment. However, as described above, the mechanical contact with grease would require a separate micro-scale model of the tribodynamic behavior. As it is not suitable for the analysis in the scale of the structural components, the paper presents experimental investigations and numerical analyses that are simplified (with respect to the micro-scale model) but quite detailed regarding contact modelling (with respect to the scale of components) of the contact behavior. 

## 2. Materials and Methods

### 2.1. Preliminary Testing

To run high-quality NVH simulations or any other dynamic investigations, it is highly recommended to perform a preliminary checkout of dynamics for the main structural components of the assembly. This can be realized with success with modal analysis.

#### Numerical and Experimental Modal Analysis

The full assembly of the EPS consists of many components such as bushings, bearings, suspensors, etc., but the main internal structural components of which the dynamic characteristics have a direct influence on the whole assembly performance are the rack and pinion. In this subsection, the selected structural mode shapes, for comparison with those experimentally identified, are presented. The numerical modal analysis was performed under free–free boundary conditions so the results represent the underlining component dynamic characteristic, which will change in the assembly due to the loads and constraints.

**Figure 3:**
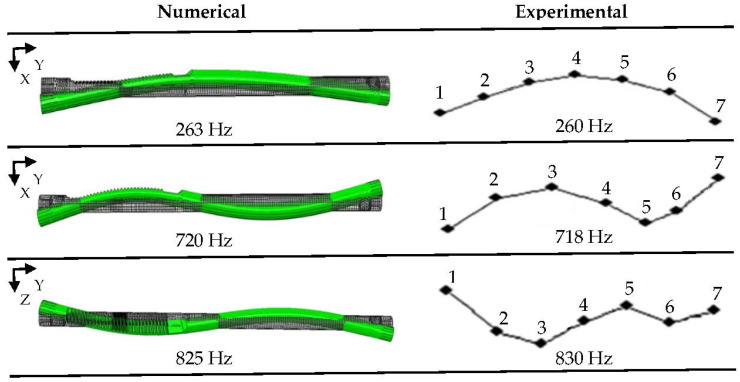
Experimental modal analysis: rack points and mode shapes.

Similar to computational modal analysis, experimental modal analysis was conducted. Approximated free-free boundary conditions were provided by hanging investigated components on low stiffness elastic bands and springs. The natural frequencies and mode shapes of the mentioned components served as a benchmark for the validation of FEM models defined for full system simulation purposes. Usual road condition excitations cover frequencies less than 50 Hz, so the considered measurement frequency range of interest was set to 0–1 kHz with 1600 spectral lines. Preliminary testing modal analysis was carried out with use of an impact hammer, which is more convenient to set up and conduct in the case of relatively small and uniform parts such as a rack, pinion or EPS housing. A miniature piezoelectric accelerometer was used for acceleration measurements at chosen points of investigated parts, and a roving accelerometer approach was implemented. Pinion and rack geometries were discretized by five and seven measurement points each. Point coordinates were used to define a simple modal geometrical model of each investigated part in order to compute mode shapes. A graphical comparison of the selected numerically and experimentally modes of the rack is presented in Figure 3.

Table 1 and Table 2 present the full list of the modes and frequencies of the rack and pinion.

High stiffness of the pinion raised problems with the identification of the experimental mode shapes (due to its complexity). However, there was good coincidence between the numerical and experimental frequencies. The preliminary modal test gave a general overview of the stiffness of the particular components and their structural modes relevance in further investigations.

### 2.2. EPS Assembly NVH Performance Investigations

#### 2.2.1. Numerical Simulations

**Figure 4:**
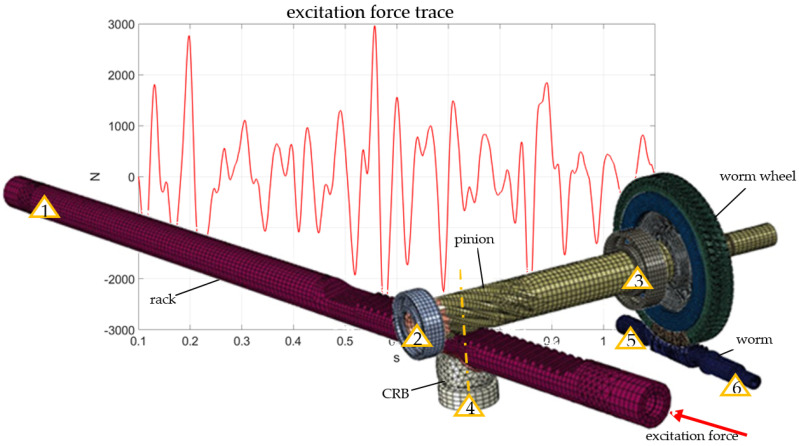
FEM model with boundary conditions.

The model created for simulations is presented in Figure 4. It consists of the rack pinion, CRB (Central Rack Bushing), pinion bearings (raceways only), and worm and worm wheel. Partially, the components and elastic supports were implemented with the use of bushing connectors (Abaqus).

The general rules for model creation such as mesh density, discrete model of the component creation, and selection of the components can be found in the full technical documentation of the analysis [35]. 

The model boundary conditions included external loads (excitation force, gravity) and the supports that corresponded to the component placements in the housing:

1. bushing support (bushing connector)
2. bearing support
3. bearing support
4. spring preload (axial movement only)
5. worm support (worm damper)
6. worm support (worm damper, power pack special element)

In Figure 4, BC’s and components related to the bearing clearances were introduced in the model via bushing connectors defined between the inner and outer bearing raceways.

The simulations were run in two steps of implicit dynamic analysis in Abaqus software. The first step was the initial preload step, and the second step was the actual simulation with the excitation force (see Figure 4). The excitation trace was a real force value measured during the real test drives on the test track. The measured excitation force was applied both in numerical and experimental tests. The initial simulations were based on the general contact with hard contact penalty properties. The component movement was “locked” by fixed rotation of the worm at point no. 6 of the support.

Detailed description of the model sensitivity and its behavior with respect to the change in the boundary conditions and interactions is presented in section Results.

#### 2.2.2. Experimental Measurements

**Figure 5:**
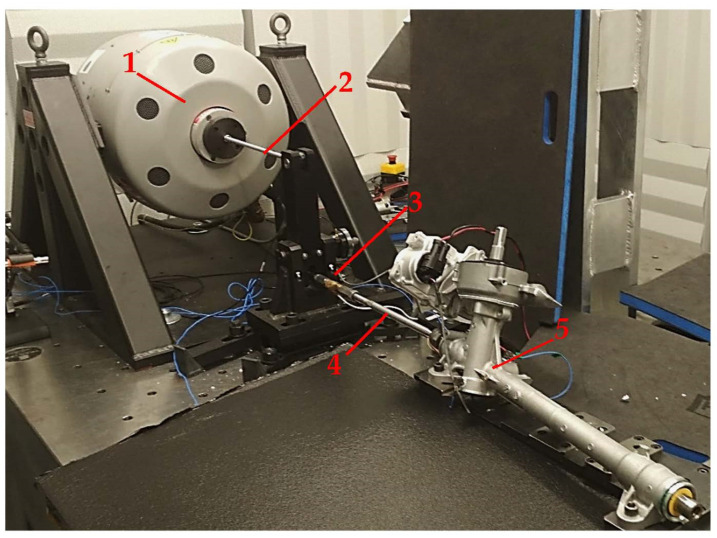
Test rig. 1—electrodynamic shaker, 2—stinger, 3—force sensor, 4—tie rod, 5—Tested EPS.

Investigations of acceleration values were needed in order to identify the influence of the rack–pinion interface on the dynamic characteristics of the EPS and FEM model fine tuning, as well as the identification of important physical phenomena that influenced the model. Measurements were conducted with the use of a field data replication technique. The EPS assembly was excited by means of an electrodynamic shaker linked to the rack end via a tie rod-mounting point. The set-up of the measurements test rig is presented in Figure 5.

**Figure 6:**
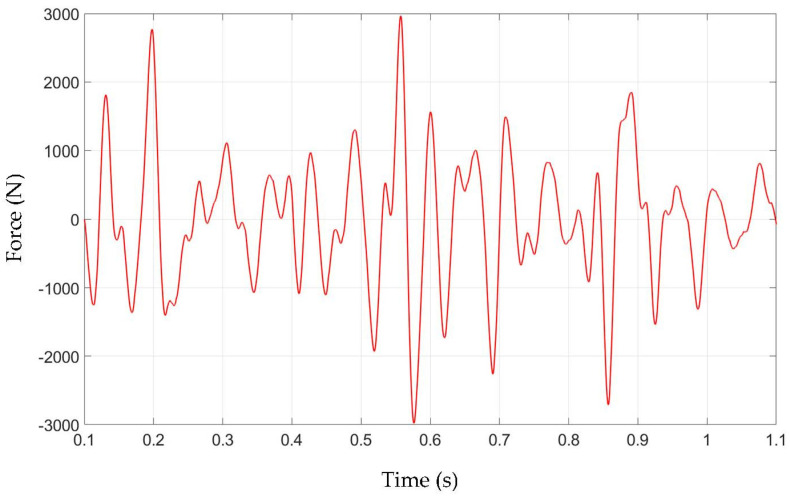
Excitation force time trace.

The excitation signal introduced to the assembly (Figure 6) was a 1-s-long force time signal recorded at the vehicle tie rod during a test drive at the track. Data acquisition, at the test rig, was started when the excitation signal and shaker output force (input to output) error were lower than 1% and the test environment was stable. The time for each measurement was at least 60 s, and the number of excitation instances was recorded in order to verify the repeatability of conducted measurements.

For general purposes, acceleration measurements were conducted at two points at the EPS housing and one point at the rack. However, the crucial measurement point was placed inside the CRB as a main point for model tuning and validation. Selection of that point was indicated by the preliminary simulations and testing. The point was located in the area of the most excessive and clear response generation and of the system. Moreover, the limitation of the CRB movement to one axis only excluded the influence of other possible signals not related to the NVH problem 

**Figure 7:**
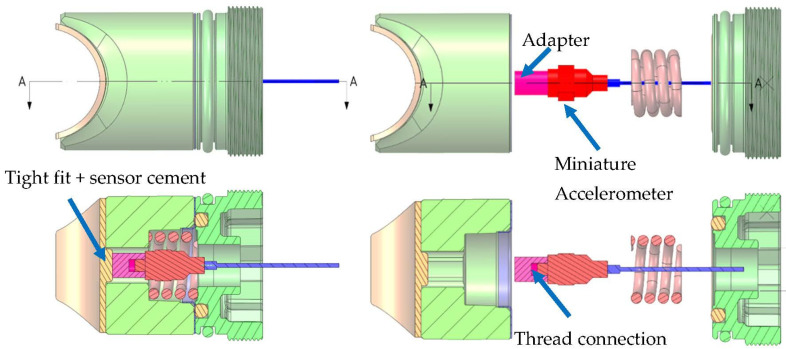
CRB accelerometer mounting arrangement.

**Figure 8:**
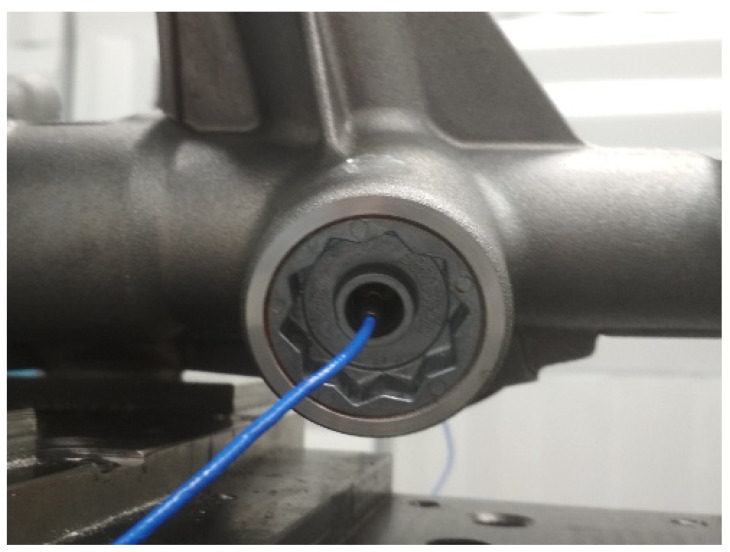
CRB measurement sensor.

A single axis accelerometer measured the vibration of the CRB in the direction perpendicular to the rack. The accelerometer placed inside the CRB assembly was firmly attached to the main body of the CRB by means of a custom adapter cemented into the body (Figure 7). The assembled sensor ready for measurements is presented in Figure 8.

All results presented in this paper show one, chosen for analysis, excitation instance that lasted 1 s, starting exactly at the starting point of the excitation signal in the case of each recorded measurement. The 1-s data samples for each recorded channel were chosen in post processing based on the force signal samples presenting one full excitation instance. 

The first set of measurements was conducted on the Electric Power Steering (Nominal Production) specimen, lubricated with molybdenum-treated lithium grease at bearing and contact surfaces. Measurements were carried under the excitation conditions mentioned above. A second set of measurements was carried out in the absence of lubrication of the rack–pinion mesh and CRB. A nominal production of the EPS specimen was disassembled and degreased in the area of the rack–pinion mesh interface. The EPS was then assembled and preloaded with 500 N at CRB. Unfortunately, it was observed that the preload introduced at CRB in the case of the non-greased mesh interface was not stable during tests and changed as soon as the rack was cycled or moved from the initial position. This was probably caused by the absence of lubrication at the mesh interface. After a full EPS control cycle, due to increased friction at the mentioned interface, the rack and pinion teeth showed a tendency to block at various points along mesh pressure line, causing changes in CRB spring displacement and thus in the nominal CRB preload value.

## 3. Results

### 3.1. Experimental Measurement Results

**Figure 9:**
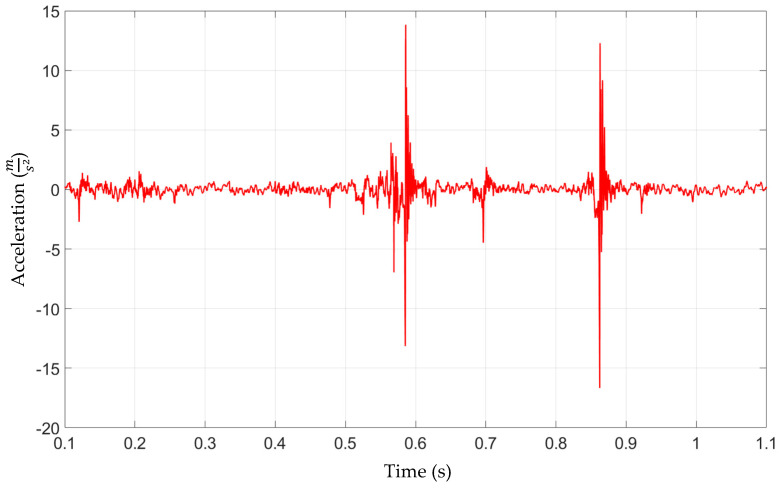
Electric Power Steering (Nominal Production)–Acceleration signal at CRB, 500 N preload.

Acceleration measurements at CRB, in the case of a nominal production specimen, including lubrication at contact interfaces are presented in Figure 9. Two district acceleration peaks of 14 m/s^2^ at and 13 m/s^2^ are visible at 0.55 s and 0.85 s of the measurement time. This indicates an impact/shock origin noise and vibration problem. A strong increase in the acceleration signal at the peak could be caused by sudden, momentary loss of contact between CRB and the rack and its rapid re-closing. The same phenomenon can occur between the rack and pinion or a combination of both.

**Figure 10:**
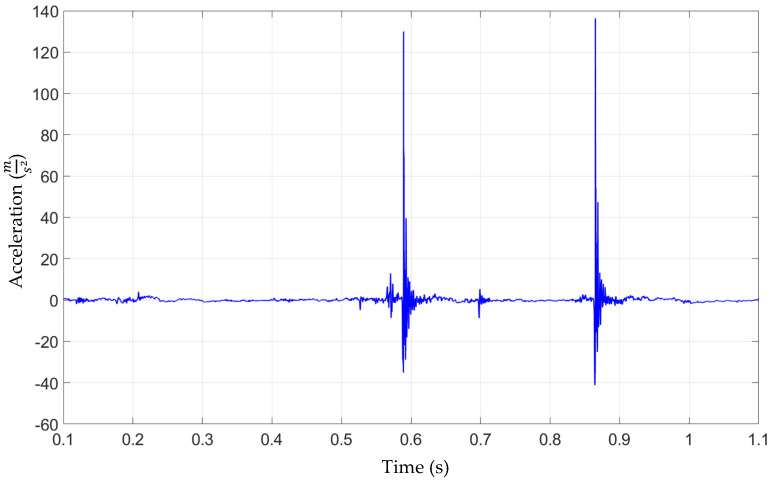
Electric Power Steering (Nominal Production)–Acceleration signal at CRB, 500 N preload, no grease at the rack–pinion and CRB interface.

Figure 10 presents acceleration measurements at CRB without lubrication at the interface. The qualitative form of the acceleration signal is similar to the production specimen results shown in Figure 9. Two district acceleration peaks are visible at 0.55 s and 0.85 s. Due to the absence of grease, the acceleration values at the peak increased significantly, up to 125 m/s^2^ in the first occurrence and up to 128 m/s^2^ in the second one. Once again, the strong increase in the acceleration signal at the peak could be caused by sudden, momentary loss of contact between EPS components due to dynamic external loading. The absence of grease at crucial system interfaces contributed to fewer energy dissipation possibilities, thus increasing the peak acceleration values.

### 3.2. Numerical Simulation Results

**Figure 11:**
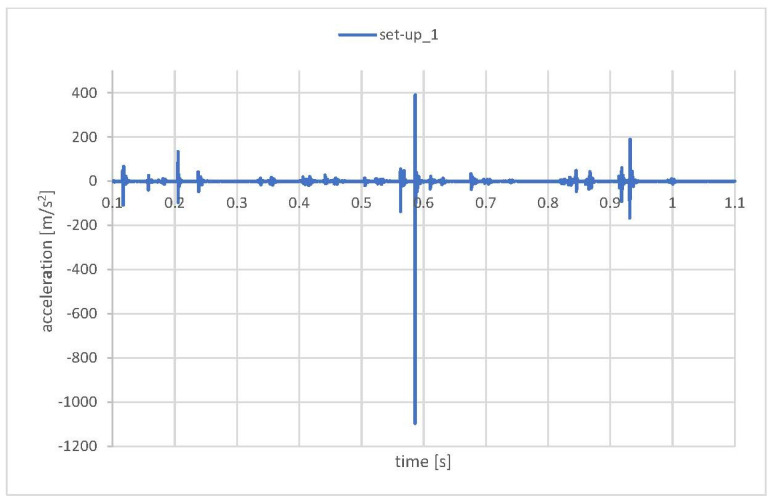
Acceleration trace in one of the initial simulations.

The numerical analyses were conducted to tune and validate the model with respect to the experimental testing. As a result, a sensitivity analysis of the model, for different boundary conditions and interactions, was performed. The first analyses were run “roughly” to mark “the starting point” and estimate the level of discrepancy between the numerical and experimental values. As a rough analysis, the authors had in mind the definition of the contact as general contact with a hard linear penalty definition (in the normal direction) and penalty contact with the friction coefficient (specific to the unique material contact pair individually) in the tangential direction. Moreover, the model was “locked” by fixed rotation at support no. 6 and the elastic support (axial motion worm dampers were not active). In all simulations presented in this paper, the load was the force trace according to Figure 6. The results of this initial analysis revealed excessive dynamic responses that were several times greater than the real one. The time trace of the acceleration on the CRB (axial direction at specified CRB node) in one of the initial simulations is presented in Figure 11.

As presented in the paper, the results of the numerical analysis started at the 0.1 s time point and lasted until the 1.1 s time point, which corresponds directly to the force trace and experimental test. The time between 0.0 s and 0.1 s is the preload step and is not relevant in the results analysis.

**Figure 12:**
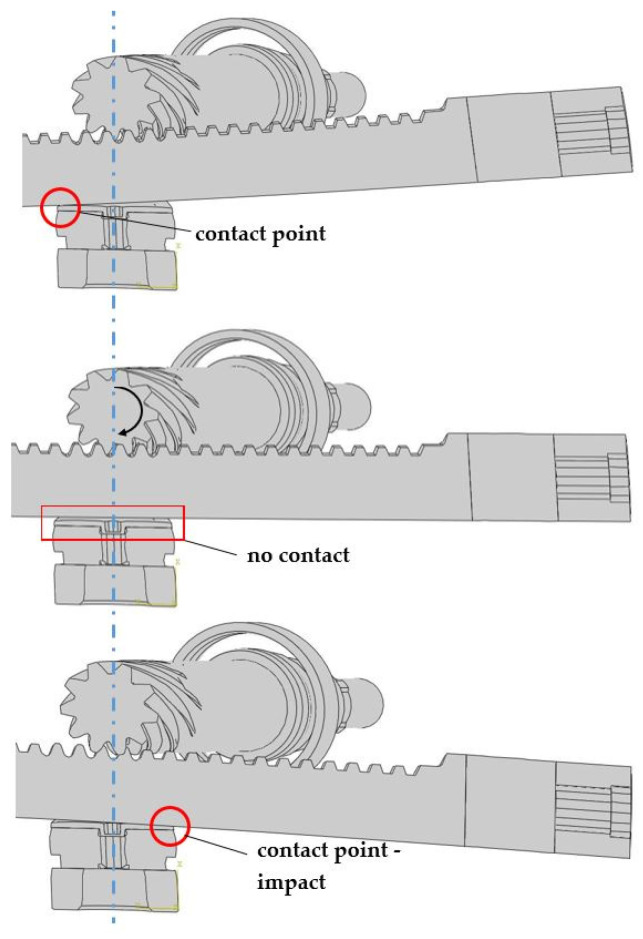
The main impact mechanism.

The accelerations were excessively high, but the behavior of the model based on the qualitative manner was correct. As expected and observed in the preliminary numerical and experimental simulations, the main excitation was in the contact pairs CRB–RACK and RACK–PINION. The character of the impact is presented in Figure 12.

As the behavior of the model was correct, but the accelerations were excessively high, further investigations took under consideration possible mechanisms of energy dissipation in the model, which could significantly reduce the impact energy between the components. The following modifications were investigated: elastic support of the worm (worm dampers), contact stiffness definitions (pressure overclosure: hard-penalty-linear; hard-penalty-nonlinear; exponential), damping of the CRB spring, and contact damping. As an additional aspect, the assumed fixed rotation of the worm was investigated. Experimental checkout of the worm transmission revealed that limited rotation of the worm was observable under the applied load. That observation changed the idea about the mechanics of the energy transfer. As the connection worm–worm wheel is a non self-locking interaction, part of the energy must be transferred and dissipated in the power-pack component, which in this case is the electric motor. The power pack was implemented in the model as an inertia element with inertia and magnetic resistance corresponding to the electric motor. The power pack was attached to the worm at supporting point no. 6 (Figure 4). The worm rotation was released and it should be stopped by the worm wheel–worm friction and power pack resistance. Several simulations were put into analysis, but no results were obtained. At the very beginning of the simulations, excessive motion of the warm was observed, which caused convergence problems and simulation crashes. As the main problem in the simulations with free worm rotation, the values of the friction coefficient in the pair worm–worm wheel and the magnetic resistance were assumed. The parameters used in the simulations were based on quasi static experimental testing. The experimentally observed behavior of the worm was high speed repetitive forward and backward rotations correlated with the trace of the loading force. Despite several trial and error attempts to justify the proper value of the coefficient of friction and magnetic resistance level under dynamic conditions, no convergence in the analysis was achieved.

It was decided to run further analyses with a fixed worm and identify the model sensitivity with respect to other changeable parameters.

**Figure 13:**
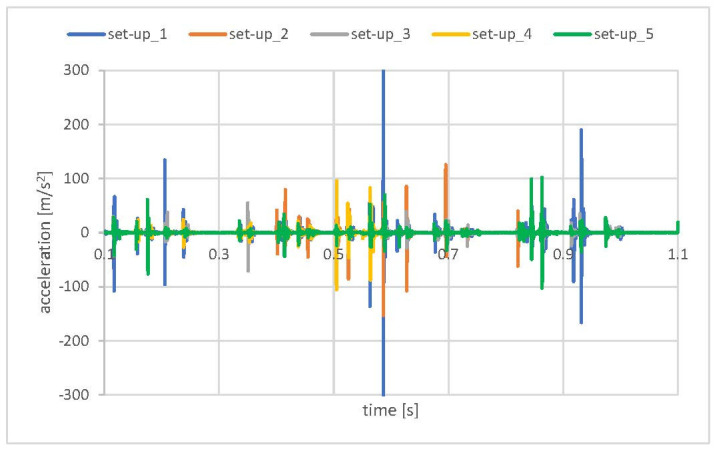
Model sensitivity analysis–CRB accelerations.

Figure 13 presents the results of analyses for the different boundary conditions/interactions. No grease presence was assumed in those simulations.

Table 3 presents the set-up of the model parameters for particular simulations.

On the basis of the performed “try and error” method, the best correlation (set-up_5) was obtained in the model with following contact definition: pressure overclosure → hard → penalty → nonlinear and viscous damping applied to the CRB preloading spring.

However, as the values of the simulations results were still far from the experimental values, the overall shock response did not exceed 60 m/s^2^; however, the incidental values greater than 100 m/s^2^ were still present, which corresponds closer to the values obtained in the test with no lubricant in the EPS. It was decided to “include” the presence of grease (which is consistent with the reality) in the impacting pair CRB–Rack in the simulation. Unfortunately no parameters of the contact with grease were accessible. The parameters of the grease, which were also not reliable, did not give any information that could be used in the simulations. Several simulation attempts with a bilinear contact damping model in pair rack–CBR were performed. Eventually, the simulation revealed a significant sensitivity to this parameter. The shock “softened” and the response (CRB acceleration) was comparable to values acquired in experimental tests.

**Figure 14:**
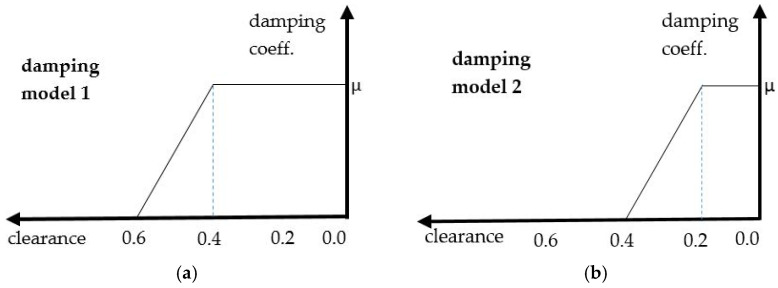
Contact damping models: (**a**) 0.6 mm total clearance; (**b**) 0.4 mm total clearance.

**Figure 15:**
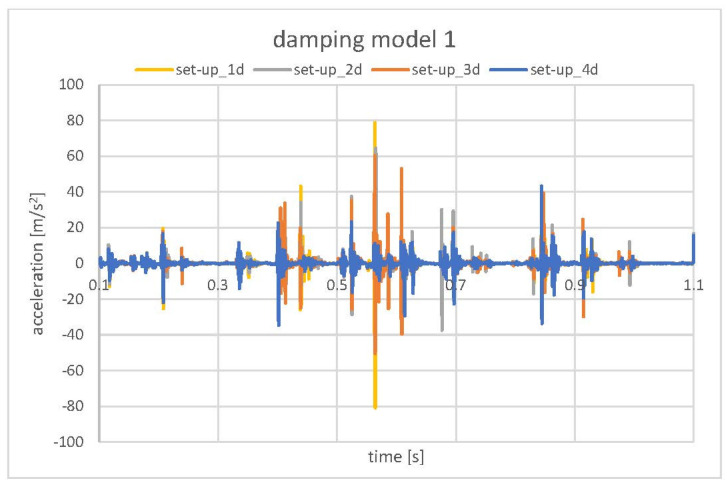
Model sensitivity analysis. Contact damping model 1–CRB accelerations.

**Figure 16:**
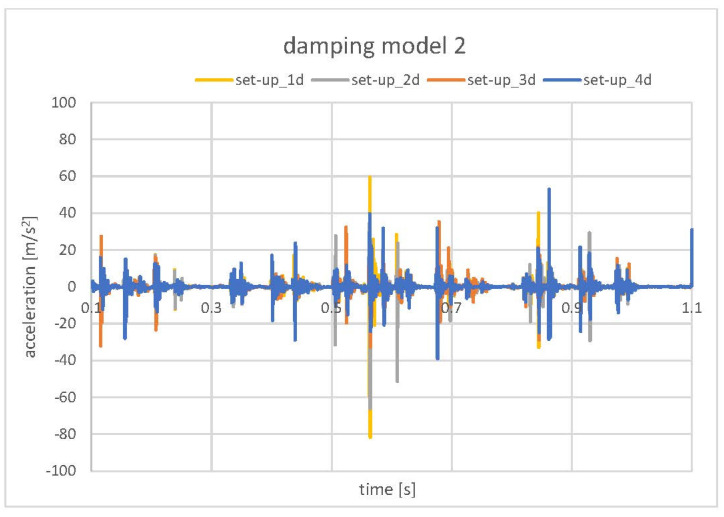
Model sensitivity analysis. Contact damping model 2–CRB accelerations.

In Figure 14, the two contact damping models that were applied to the pair CRB–Rack are presented. Model 1 represents the 0.6 mm total clearance (assumed grease layer), and model 2 represents the 0.4 mm total clearance. In both damping models, analysis of the model sensitivity was conducted with decreasing viscous damping (μ) from 0.2 to 0.05 (Table 4). In the decrease of the viscous damping coefficient value results with softening of the “damper”, one can observe for both damping models, a gradual decrease in the acceleration value of CRB (Figure 15 and Figure 16), although the tendency was more regular (damping of all of the particular impacts) in the case of model 1.

## 4. Discussion

Analysis and comparison of the numerical and experimental data indicated that the most important factors of the numerical analysis were related to the contact stiffness. A rough numerical model with the default parameters (Figure 11) significantly exceeded all of the experimentally obtained values. 

The numerical model set-up_5 (Figure 13) neglecting presence of the grease in the system indicates a general acceleration level comparable to the experimental values obtained during the test in which the lubricant was removed from the EPS assembly. However, the lowest acceleration values were observed for set-up_3 where no damping in the CRB preload spring was present. This may indicate that resistance in the movement of the CRB (friction between the CRB O-ring and housing) may be included in the model to obtain the proper mechanical behavior of the system. Nevertheless, in all of the model set-ups, the results exceeded the accelerations of standard EPS (grease presence) (Figure 9).

Finally, the models with contact damping (Figure 14), implemented to simulate the grease influence, gave the results that corresponded the closest to the standard EPS. The experimentally observed values were in the range of −15 m/s^2^ to 15 m/s^2^. The lowest values obtained in the numerical model (set-up_4d Figure 15) oscillated in a similar range. Only one pick exceeded that range and reached the value of −40 m/s^2^ to 40 m/s^2^. Due to that fact that the exact final value of the acceleration level may be the result of a specific combination of all mentioned, tested, as well as neglected in the simulation factors, the obtained results were accepted as successful at this stage of the research. The authors see the need for further, more detailed analysis; however, the main factors influencing the model sensitivity and its influence on the correlation with experimental results were defined. 

## 5. Conclusions

The results of the presented investigations led to the conclusion that in the case of an NVH analysis regarding phenomena of shock/impact characters, the parameters of contact used in the static, quasi-static or low velocity analysis are not valid. 

The first observation focuses on the contact stiffness value. In case where the impact is the phenomenon defining the analysis results, a proper definition of local stiffness is required. The observation, based on the presented simulations and measurements, leads to the conclusions that the “default” linear contact stiffness does not correspond to the actual contact behavior, by being too stiff, which results in excessive acceleration responses. Application of the nonlinear contact stiffness, where initial stiffness is low and exponentially increases with the overclosure (penalty method), indicates better accuracy for the experimental observations, by reduction of the acceleration response. However, as presented in this paper, the authors were not able to tune the model to the values of acceleration measured experimentally just by means of nonlinear contact stiffness. As a consequence, the bilinear contact damping model was applied to substitute the grease presence in the real system. The obtained results indicated a significant drop of the acceleration value.

On the basis of the presented material, the general conclusions can be formulated that for NVH analysis where the shock phenomenon is considered, the default linear penalty contact model is too stiff to give excessive model responses. A nonlinear contact model should be used to define more representative numerical models. However, in the mechanical system in the presence of grease, nonlinear contact stiffness does not fully correspond to measurement outcomes. The authors recommend the application of contact damping to include energy dissipation caused by grease. One has to remember that many of the mentioned factors used in the presented simulations did not have an experimentally proven value and were applied to observe model behavior and the potential application of the contact/damping model in NVH simulations. A lack of those values is a significant problem in NVH analyses. Nevertheless, the paper presents how to prepare a structural-scale model in which the crucial influence on the component behavior is the presence of the lubricant-material, which is “present” at the microscale level but cannot be included, by its direct modelling, which would exclude the usability of the structural model. The direction in the development of the NVH analyses should focus on research of the parameters and factors that would be reliable in dynamic analysis, as the article proved, many of those use in static analyses are not relevant.

## Figures and Tables

**Table 1:** Numerical modal modes.

Component	Frequency [Hz]									
	Mode 1	Mode 2	Mode 3	Mode 4	Mode 5	Mode 6	Mode 7	Mode 8	Mode 9	Mode 10
Rack	263	300	718	825	1413	1595	2278	2361	2601	3436
Pinion	2517	2553	5345	5413	8568	8714	8777	13,137	13,427	13,491

**Table 2:** Experimental modal modes.

Component	Frequency [Hz]		
	Mode 1	Mode 2	Mode 3
Rack	260	720	830
Pinion	2520	2540	>5 k

**Table 3:** Undamped contact simulation set-up.

Set-Up	Parameter			
	Contact	Worm Axial Support	CRB Viscous Spring Damping	Worm Rotation
1	penalty linear	fixed	no	fixed
2	penalty linear	elastic	no	fixed
3	penalty nonlinear	elastic	no	fixed
4	penalty nonlinear	elastic	0.1	fixed
5	penalty nonlinear	elastic	0.2	fixed

**Table 4:** Damped contact simulation set-up.

Set-Up	Parameter				
	Contact	Worm Axial Support	CRB Viscous Spring Damping	Worm Rotation	Damping Coefficient μ
1d	penalty linear	elastic	no	fixed	0.2
2d	penalty linear	elastic	no	fixed	0.15
3d	penalty linear	elastic	no	fixed	0.1
4d	penalty linear	elastic	no	fixed	0.05

## Data Availability

The data presented in this study are available on request from the corresponding author.

## References

[1] Dyer G. (1997). Analysis of Energy Consumption for Various Power Assisted Steering Systems. SAE Trans..

[2] (2020). Nexteer, Nexteer Supplies 40 Millionth Electric Power Steering System. https://www.nexteer.com/release/nexteer-supplies-40-millionth-electric-power-steering-system/.

[3] Schneider M., Wilhelm M., Alt N. (1995). Development of Vehicle Sound Quality-Targets and Methods. SAE Trans..

[4] Huang H.B., Wu J.H., Huang X.R., Ding W.P., Yang M.L. (2020). A novel interval analysis method to identify and reduce pure electric vehicle structure-borne noise. J. Sound Vib..

[5] Hartmann R., Holbrook G., Politano G. (1988). Starting Motor Cranking Sound-Correlation of Objective Noise Measurements to Subjective Jury Ratings.

[6] Kuroda O., Fujii Y. (1988). An Approach to Improve Engine Sound Quality.

[7] Sachs M., Brackett S. (1989). Subjective Evaluation of Simulated Engine Induction Noise.

[8] Nishio Y., Kohama T., Kuroda O. (1991). New Approach to Low-Noise Air Intake System Development.

[9] Sacks M., Hackney S. (1988). Performance of Acoustic Components for Engine Induction Systems.

[10] Busch G., Maurell R., Meyer J., Vorwerk C. Investigations on Influence of Engine Block Design Features on Noise and Vibration. Proceedings of the 1991 Noise & Vibration Conference.

[11] Dunne G., Wheeler A., Jennings P. The identification of powertrain sound quality target sounds. Proceedings of the 29th International Congress and Exhibition on Noise Control Engineering.

[12] Otto N., Amman S., Eaton C., Lake S. (1999). Guidelines for Jury Evaluations of Automotive Sounds.

[13] Brandl F., Biermayer W. (1999). A New Tool for the Onboard Objective Assessment of Vehicle Interior Noise Quality.

[14] Schulte-Fortkamp B., Genuit K., Fiebig A. (2007). A New Approach for Developing Vehicle Target Sounds. Sound Vib..

[15] Genuit K., Schulte-Fortkamp B. (2005). The acoustical design of vehicles: A new tool for benchmarking and target sound. J. Acoust. Soc. Am..

[16] Albert A., Schwarz A. (2011). Automated Identification of NVH-Phenomena in Vehicles.

[17] Chen Y., Feng H. (2021). An Overview of NVH Problems Caused by Changes in the Excitation Source of HEV. IOP Conf. Ser. Mater. Sci. Eng..

[18] Putner J., Arsic D. (2018). Investigation of NVH Phenomena at Electric Vehicles. ATZextra Worldw..

[19] Lennström D., Lindbom T., Nykänen A. (2013). Prominence of Tones in Electric Vehicle Interior Noise. Internoise 2013: Noise Control for Quality of Life.

[20] Huang H.B., Wu J.H., Huang X.R., Yang M.L., Ding W.P. (2020). A generalized inverse cascade method to identify and optimize vehicle interior noise sources. J. Sound Vib..

[21] Qian K., Hou Z., Liang J., Liu R., Sun D. (2021). Interior Sound Quality Prediction of Pure Electric Vehicles Based on Transfer Path Synthesis. Appl. Sci..

[22] Hua X., Thomas A., Shultis K. (2021). Recent progress in battery electric vehicle noise, vibration, and harshness. Sci. Prog..

[23] Kwon S., Jeon J., Park J., Lee H. (2022). Quantification of rattle noise generations from automotive compartments by variational mode decomposition. J. Sound Vib..

[24] Ma C., Chen C., Liu Q., Gao H., Li Q., Gao H., Shen Y. (2017). Sound Quality Evaluation of the Interior Noise of Pure Electric Vehicle Based on Neural Network Model. IEEE Trans. Ind. Electron..

[25] Lee J., Lee S., Kwak Y. (2015). Temporal and spectral characteristics of BSR noises and influence on auditory perception. J. Mech. Sci. Technol..

[26] Rahnejat H. (2010). Tribology and Dynamics of Engine and Powertrain.

[27] Zheng X., Luo X., Qiu Y., Hao Z. (2020). Modeling and NVH Analysis of a Full Engine Dynamic Model with Valve Train System. Appl. Sci..

[28] Mohammadpour M., Theodossiades S., Rahnejat H. Tribo-Dynamics of Differential Hypoid Gears. Proceedings of the ASME 2013 International Design Engineering Technical Conferences & Computers and Information in Engineering Conference IDETC/CIE 2013.

[29] Brecher C., Brumm M., Knecht P. Improvement of the excitation behavior of bevel gears considering tolerance fields caused by manufacturing and assembly processes. Proceedings of the International Gear Conference 2014.

[30] Billenstein D., Glenk C., Diwisch P., Rieg F., Schumacher A., Vietor T., Fiebig S., Bletzinger K.U., Maute K. (2018). Investigation of Contact Settings on the Result of Topology Optimization to Avoid Contact Stiffness Supports. Advances in Structural and Multidisciplinary Optimization.

[31] Ling X. (2016). Contact Stiffness Estimation Based on Structural Frequency Response. U.S. Patent.

[32] Stein E., de Borst R., Hughes T.J.R. (2004). Encyclopedia of Computational Mechanics. Volume 2: Solids and Structures.

[33] Laursen T.A. (2003). Computational Contact and Impact Mechanics.

[34] Kim J.-T., Lee J.W., Lee S.M., Lee T., Kim W.-G. Study on Mechanism of Impact Noise on Steering Gear While Turning Steering Wheel in Opposite Directions. Proceedings of the Inter-Noise Conference.

[35] Fiebig W., Pietrusiak D., Wilhelm J., Wróbel J. (2019). UKL Steering Gear Vibrations Testing and Modelling. Faculty of Mechanical Engineering Wroclaw University of Science and Technology Report.

